# Strengthening health technology assessment (HTA) in the European Union: insights from Slovenia’s implementation journey

**DOI:** 10.1017/S0266462325103383

**Published:** 2026-02-16

**Authors:** Katarina Beravs-Bervar, Wim Goettsch, Iñaki Gutierrez-Ibarluzea, Adam Skali, Alric Rűther, Iga Lipska, Eva Turk

**Affiliations:** 1 Slovenian Quality Health Care Agency, Slovenia; 2 Universiteit Utrecht, Netherlands; 3 Healthcare Institute of the Netherlands, Netherlands; 4 Directorate of Health Research, Innovation and Evaluation, Ministry for Health, Spain; 5 Institute for Human Centered Health Innovation, e-Health Innovation Center, Morocco; 6 International Affairs, Institut fur Qualitat und Wirtschaftlichkeit im Gesundheitswesen, Germany; 7 Institute for Health Policy, Poland; 8 Center for Competence Development, Integrated Care and e-Health, Medical University of Gdańsk, 80-210 Gdańsk, Poland; 9 Ministry of Health of the Republic of Slovenia, Slovenia; 10 Medical Faculty, University of Maribor, Slovenia

**Keywords:** HTA Implementation, HTA process, HTA Regulation, Slovenia, Stakeholder engagement, Multi-stakeholder

## Abstract

**Introduction:**

Slovenia has engaged with Health Technology Assessment (HTA) for over two decades, but its system remains fragmented and underdeveloped. Until recently, responsibilities for evaluating health technologies were dispersed across multiple institutions without a central coordinating body or standardized methodology. Medicinal products have been subject to structured evaluation through the Health Insurance Institute of Slovenia, while other health technologies, including medical devices, diagnostics, and preventive interventions, have followed less consistent pathways under the Ministry of Health. The adoption of the European Union Health Technology Assessment Regulation), entering into force in January 2025, has provided new impetus for reform, requiring Slovenia to designate a national HTA body to participate in joint clinical assessments and align national processes with EU standards.

**Methods:**

A mixed-methods analysis combining a narrative overview of HTA in Slovenia with findings from two multi-stakeholder workshops held in 2025. These workshops, which convened Slovenian and international experts, policymakers, clinicians, and patient representatives, explored opportunities and challenges for developing a robust HTA framework.

**Results:**

Key findings highlight the need to strengthen methodological capacity, introduce systematic stakeholder engagement, ensure transparency, and integrate real-world evidence into decision-making. Particular emphasis was placed on expanding HTA to medical devices, diagnostics, and digital health technologies, and on anticipating future innovations such as artificial intelligence.

**Conclusions:**

Slovenia now stands at a pivotal juncture. Establishing a central HTA body with a clear legal mandate, building national expertise, and leveraging regional and European collaboration is essential to creating a transparent, evidence-based, and patient-centred HTA system.

## Introduction

Slovenia initiated its engagement with health technology assessment (HTA) in response to a growing recognition of the need for structured, evidence-based evaluation of health technologies to inform healthcare decision-making. Over the past 25 years, Slovenia has lacked a unified methodological framework for integrating diverse health technologies into the publicly funded healthcare system. No formal decision was reached regarding which institution should serve as the national focal point for HTA – whether the Agency for Medicinal Products and Medical Devices (JAZMP), the Health Insurance Institute of Slovenia (HIIS), the National Institute of Public Health (NIJZ), or the Ministry of Health (MoH). In the absence of a designated HTA authority, Slovenia opted instead to prioritize international collaboration. By joining EUnetHTA (European Network for Health Technology Assessment), Slovenia gained access to valuable expertise, methodologies, and peer exchange, laying important groundwork for the development of its national HTA framework (1). Although procedural pathways for new applicants to be considered for benefit package and thus, previous HTA evaluation, have been established, they lack a consistent methodological foundation and standardized criteria applicable across all health technologies. Furthermore, the availability of skilled personnel and the dissemination of HTA-related expertise remain limited. The medicinal products are most stringently regulated and evaluated domain in the context of HTA. The integration of medicinal products into public funding is managed by the Pharmaceutical Reimbursement Committee, an advisory entity to the HIIS. This committee formulates its recommendations based on the procedures outlined in the *Rules on the Inclusion of Medicines in the List* (2). In contrast, other health technologies – such as diagnostic and therapeutic procedures, as well as organizational innovations (e.g., telemedicine, digital health systems), vaccination programs, screening initiatives, and other preventive or curative interventions – are subject to significantly less regulation and evaluation and exhibit greater procedural ambiguity in HTA. These technologies are evaluated by the Health Council, the principal advisory body to the MoH, in accordance with the *Procedures on Handling Applications for New Healthcare Programs* (3).

Hence, Slovenia is in urgent need of a more structured HTA system to ensure transparent, evidence-based decision-making in healthcare, including the role of stakeholders (patients, professionals, managers, and citizens) and the roadmap to decision. While significant steps have been taken in the past decade, including international collaborations, the national framework remains fragmented, and underdeveloped.

The urgency for reform was sparked by the new EU Regulation on Health Technology Assessment (Regulation (EU) 2021/2282), which entered into force in January 2022, and has been applied since January 2025 (4). This regulation mandates the involvement of national HTA bodies in joint clinical assessments (JCA) at the European level for certain categories of medicines and medical devices. Slovenia, like other member states, is required to designate a formal HTA body or organization (different approaches have been established in other countries) capable of participating in EU-level assessments and integrating European outputs into national decisions.

In response, the Slovenian Law on Quality in Healthcare introduces the legal basis for the creation of a national HTA agency or coordinating body (5). This institution will serve as the central authority for coordinating HTA in Slovenia, aligning national procedures with EU requirements and international best practices, and ensuring consistency, transparency, and methodological robustness across all types of technologies. Such a body would ideally coordinate HTA across ministries, agencies, and payer systems, include systematic processes for horizon scanning, real-world data collection and reassessment, involve patients and healthcare professionals in assessments, maintain transparent criteria and publish assessment outcomes, support innovation through clear pathways for market entry and evaluation, build national expertise in HTA methods, including those suited for digital and cross-sectorial technologies, act as the Slovenian contact point for EU HTA cooperation and joint assessments.

This paper aims to contribute to the development of a robust HTA system in Slovenia by examining the current state of HTA in the country, identifying the necessary changes required to align it with the EU HTAR, and outlining recommendations for the future.

## Methods

This paper adopts a mixed-methods approach. It first presents a narrative overview of the current state of HTA in Slovenia and developments at the EU level. It then incorporates findings from a two multi-stakeholder workshops held in Slovenia, which brought together Slovenian and international experts, policymakers, clinicians, and patient representatives to discuss opportunities and challenges for HTA implementation.

The first 2-day multi-stakeholder event titled **“HTA Regulation is Here, What Now?,”** aimed at exploring the implications of the newly implemented Health Technology Assessment Regulation across Europe. Originally envisioned as a local gathering focused on engaging domestic stakeholders, the event evolved into an international forum, drawing over 80 participants from Slovenia as well as Croatia, Hungary, Slovakia, and the Czech Republic. In addition, prominent plenary speakers and workshop facilitators from Ireland, Germany, the Netherlands, Spain, Poland, and Italy (see Supplementary Material on agenda and contributors), contributed their expertise, enriching the discourse with diverse perspectives and cross-border insights in the form of keynote lecture, panel discussions and workshops.

The second multi-stakeholder event was focused on medical devices and attracted over 30 participants from the field. Organized as a workshop of thematic stations, each station convened six to seven participants with diverse expertise, ensuring a comprehensive examination of HTA dimensions from multiple stakeholder perspectives, was a first event organized to solely focus on medical devices and HTA.

## Results

### Narrative overview: current state of HTA in Slovenia

**Figure 1.**
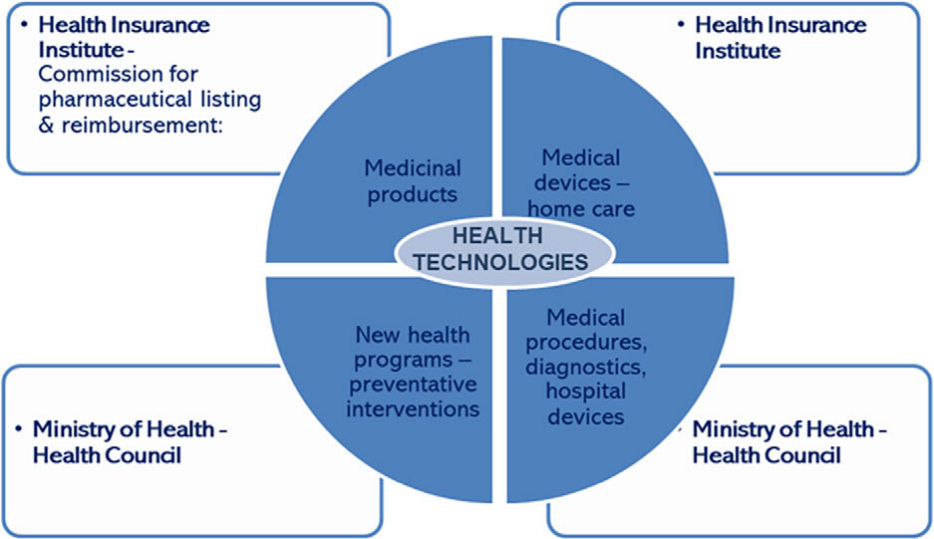
Slovenia’s HTA process.

Slovenia’s HTA processes remain fragmented, with responsibilities divided across multiple bodies. Medicinal products are primarily assessed by the HIIS, which applies structured criteria including safety, clinical efficacy, cost-effectiveness, and budget impact. Health technologies, such as hospital medical devices, diagnostics, and preventive interventions, are evaluated through the Health Council, though processes are less standardized and lack methodological consistency (see Figure 1).

Despite the establishment of procedural pathways, Slovenia lacks a central HTA institution with a legal mandate to oversee and coordinate all assessments. Human resources and expertise in HTA remain limited. The EU HTAR, entering into force in 2025, requires Slovenia to designate such a body, creating both a challenge and an opportunity for systemic reform.

### Workshop findings

The workshop (see agenda in Supplementary Material) explored HTA development in Slovenia under three main themes. Participants stressed the importance of incorporating identification of gaps and needs that feeds early HTA dialogues with professionals and innovators, horizon scanning, and disinvestment strategies. They highlighted the need for structured processes that follow the entire lifecycle of health technologies, from innovation to withdrawal, supported by real-world evidence. Stakeholders recognized the necessity of adapting Slovenian HTA processes to meet EU requirements, particularly for JCAs. The discussion underlined the importance of transparency, methodological quality, and trust in transnational exchanges. The workshop also emphasized the value of hospital-level perspectives in HTA including those issues related to implementation in different contexts. Exercises on developing PICO (Population, Intervention, Comparison, Outcomes) questions illustrated how structured frameworks can strengthen national assessments and ensure alignment with EU-level methodologies. Key takeaways from the workshop were that quality, transparency, and trust are essential for effective HTA cooperation, Slovenia should prioritize capacity-building and stakeholder engagement, and lessons from other countries can inform pragmatic national solutions.

The following key takeaways from the event are important to consider when taking the next steps of HTA development in Slovenia. The actions stemming from the learnings should be executed concurrently rather than sequentially. This approach will enable faster progress and foster the synergistic development of various elements, allowing for lessons to be learned from any failures that may arise:

- **Strengthening capacity and collaboration** – As HTA agencies refine or expand, there is a consistent need for well-trained professionals and clear stakeholder collaboration. Governments, industry, clinicians, and patient groups must recognize each other’s roles and maintain open lines of communication. Partnerships can also help smaller or newer HTA programs leverage external expertise until they develop sufficient internal capacity.
- **Emphasizing the patient perspective** – The significance of patient-reported outcomes and real-world experiences is widely recognized. Ongoing education and engagement of patient groups remain crucial for shaping relevant research questions and interpreting evidence in a practical context, including the management of expectancies when judging value of technologies based on fragmented and diverse quality of data.
- **Anticipating future innovations** – Rapid technological change – including AI, digital health tools, and novel treatments – demands that HTA frameworks stay agile. - “Living” HTA models and advanced data analytics could transform how assessments are conducted, encouraging continuous improvements in healthcare decision-making.

Though the transition toward more inclusive, dynamic, and methodologically balanced HTA processes poses challenges, panelists were optimistic that sustained collaboration and gradual adaptation will enhance healthcare quality and patient outcomes. Independence of HTA agencies and structured patient engagement, both in questions and outcomes definition and the elaboration of recommendations for decision (deliberative processes), will likely remain cornerstones in building credible, transparent, and relevant assessment system.

The workshop on medical devices (MDs) and in vitro diagnostic medical devices (IVDs) (see agenda in Supplementary Material) reaffirmed the need to establish a robust HTA framework for MDs and IVDs, grounded in legal and institutional infrastructure, standardized assessment methodologies, data utilization, continuous stakeholder training, and adaptive capacity – especially considering emerging digital innovations:

- **Integration of non-clinical dimension**s: participants emphasized the importance of incorporating non-clinical dimensions – economic, organizational, social, ethical, environmental, and legal – into the HTA of MDs and IVDs. Key priorities included the clearer delineation of the organizational dimension, structured stakeholder engagement (e.g., distributors), application of the PICO framework, and integration of both qualitative and quantitative methodologies. Recommendations encompassed the establishment of a legal basis, defined timelines, and transparent criteria for the selection of technologies for assessment and reassessment.
- **Institutional coordination and multi-criteria evaluation:** given the multidimensional nature of medical device evaluation, a multi-criteria approach was advocated. It was proposed that the newly established National Agency for Quality in Care assume a formalized role as coordinator, standard-setter, and advisor within a legally defined mandate.
- **Standardization of procedures and capacity building:** on procedural aspects, participants called for transparent methodologies, standardized guidelines and templates, and independent evaluations. Stakeholder involvement should be formalized, with the agency maintaining an advisory function. The necessity of systematic expert training and periodic reassessment (every 3–5 years), when substantial evidence has been published, was underscored.
- **Performance measurement and data utilization:** the discussion also highlighted the importance of defining key performance indicators and utilizing existing data sources to assess technology safety and effectiveness. Broad stakeholder inclusion – particularly of patients – and regular technology use reporting were identified as priorities.
- **Digital health and AI integration:** the final session addressed digital health and artificial intelligence (AI), noting the low digital maturity. While AI’s potential was acknowledged, concerns were raised regarding ethical considerations, transparency (white-box versus black-box models), and decision accountability. Effective integration of AI into HTA processes will require robust governance structures ensuring clarity and oversight.

## Discussion

Slovenia’s HTA system is at an early stage, but progressing rapidly due to EU-driven reforms. The creation of a national HTA unit in 2024 and legal backing through the Law on Quality in Healthcare provide a foundation for institutional development. However, substantial challenges remain, including limited human resources, methodological gaps, and institutional fragmentation.

To align with the EU HTAR, Slovenia must undertake a series of strategic reforms, beginning with the establishment of a central HTA agency endowed with a clear legal mandate and operational independence. Building national capacity in HTA methodologies is essential, particularly in areas such as economic evaluation, multicriteria and multidimension analysis, comparative effectiveness research, and digital health assessment. A deeper examination of various elements of the HTA process in Slovenia – such as prioritization, PICO formulation, and the role of HTA in decision-making – along with institutional challenges balancing national requirements and the common good, will be essential for fostering mutual understanding and ensuring effective implementation of the HTA process. This will require investment in training, institutional infrastructure, and cross-sector collaboration. Equally important is the systematic inclusion of stakeholders – especially patients and clinicians – whose insights are critical for ensuring that HTA processes are contextually relevant and ethically grounded. Furthermore, the development of standardized, transparent, and evidence-based procedures for the assessment of other health technologies, including medical devices and diagnostics, will enhance the robustness and credibility of the national HTA system.

Several countries in Central and Eastern Europe offer valuable examples for Slovenia. Poland’s AOTMiT has developed comprehensive HTA guidelines that support transparent and evidence-based decision-making for both medicinal and other health technologies (6). Hungary has built a strong academic foundation for HTA, with a dedicated Department of HTA and active international collaboration, and has published a roadmap for HTA implementation that emphasizes capacity building and methodological rigor (7). Czechia has made progress in institutionalizing HTA and has emphasized regional cooperation and standardization of methodology, particularly in the context of digital health and pharmaceutical evaluations (8). The examples above show the importance of tailoring HTA systems to national contexts while leveraging regional collaboration to accelerate implementation and avoid inefficiencies. It is worth noting that Slovenia has a population of around 2 million inhabitants and that in comparison to other bigger countries in Europe, the decentralization and fragmentation of HTA processes make no sense, as it would affect the quality of the outputs in an environment of scarcity of skilled human resources, specifically in certain required areas of knowledge.

## Limitations

This was the first instance of organizing multi-stakeholder events in Slovenia aimed at fostering inclusive HTA dialogue across the health ecosystem. Despite extending invitations to all relevant stakeholder groups, the representation of patient advocates and clinical professionals was notably lower than other groups. This imbalance may have influenced the diversity of perspectives captured during the events and highlights the need for targeted engagement strategies to ensure more equitable participation in future initiatives. However, the conclusions of the workshops precisely remarked and highlighted the need for patients and clinicians’ involvement in the process and their pivotal role in certain phases of the process, such as questions and outcomes definition and deliberation.

## Conclusions and recommendations

HTA in Slovenia is evolving, but it requires significant strengthening to meet both national needs and EU obligations. Based on the narrative overview and recent workshop findings, several priorities emerge. Slovenia should formally establish a national HTA body with a clear mandate to coordinate and oversee all HTA activities, which has been done under the Slovenian Quality Health Care Agency. To ensure sustainability, investments in training HTA professionals and building methodological expertise are needed, supported by EU initiatives such as the technical support instrument (TSI). Slovenia is also actively participating in the HTAR Capacity Building Programme Virtual Classrooms, organized by the HAG Insight Consortium.

At the same time, structured mechanisms for involving patients, clinicians, technology developers, and payers must be introduced to ensure inclusiveness and legitimacy of assessments. Developing clear guidelines and standardized templates for evaluating both medicinal and other health technologies will provide consistency and transparency. Slovenia would also benefit from regional collaboration, engaging with other Central and Eastern European countries, specifically those of similar distribution and inhabitant size, facing similar challenges to exchange experiences and best practices.

Looking ahead, the system should be future-oriented, incorporating digital health and artificial intelligence into HTA frameworks while safeguarding ethical standards and transparency. The State of Play report for Slovenia, currently being prepared under the TSI, could serve as a valuable baseline for reform and an anchor for strategic development.

Slovenia now stands at a pivotal point in its HTA journey. With coordinated efforts, clear priorities, and strong international collaboration, the country has the potential to build a robust, transparent, and patient-centered HTA system aligned with European standards. Strengthening ties with European and global HTA partners will not only help Slovenia address its national challenges but also generate shared benefits and synergies for all stakeholders across Europe.

## Supporting information

10.1017/S0266462325103383.sm001 Beravs-Bervar et al. supplementary material Beravs-Bervar et al. supplementary material

## Supplementary material

The supplementary material for this article can be found at http://doi.org/10.1017/S0266462325103383.
