# Supplementary material for: Strengthening health technology assessment (HTA) in the European Union: insights from Slovenia’s implementation journey
Source: Int J Technol Assess Health Care. 2026 Feb 16;42(1):e23. doi: 10.1017/S0266462325103383 (PMC12979015; doi:10.1017/S0266462325103383)
Supplement: Beravs-Bervar et al. supplementary material [file S0266462325103383sup001.pdf]

European Commission
REPUBLIC OF SLOVENIA
MINISTRY OF HEALTH

Day 1, 11. 2. 2025: High-Level Talks and Panel Discussions

8:30 – 9:00: Arrival and registration of participants

9:00 – 9:45: Opening Remarks

- Welcome by the Event Chair: **Eva Turk**, MoH Slovenia
- Opening speech**: Denis Kordež, State Secretary at MoH Slovenia**
- Overview of the Event Agenda: **Eva Turk**, MoH Slovenia

9:45 – 10:15: Keynote Address

- Speaker: **Roisin Adams,** Chair of the EU Member State HTA Coordination group

10:15 – 12:00: Panel Discussion

Innovations in HTA Methodologies

- Moderator: **Eva Turk**, MoH Slovenia
- Panelists: – **Iga Lipska**, Pomeranian Hospitals, Poland
  – **Inaki Gutierrez-Ibarluzea**, MoH Basque County, Spain
  – **Wim Goetsch**, Utrecht University, Netherlands
  – **Marco Marchetti**, AGENAS, Italy

12:00 – 13:00: Lunch Break

13:00 – 17: 00: Parallel Workshops (Day 1)

	**Workshop A:** Evidence informed decision making in health care organizations	**Workshop B:** Innovation and Lifecycle Approach (including Early HTA)	**Workshop C:** Rapid assessments	**Workshop D:** HTAR and implementation
**Workshop leader**	**Iga Lipska**	**Inaki Gutierrez-Ibarluzea and Michal Stanak**	**Wim Goetsch and Alric Ruether**	**Marco Marchetti and Aydin Burçak**
13:00 – 15:00 Parallel Workshops<br>15:00 – 15:30 **Networking break**<br>15:30 – 17:00 Continued Workshops	• Focus on implementing HTA in hospital settings<br>• Case studies and group discussions	• Strategies for assessing health technologies throughout their lifecycle<br>• Interactive discussions on early HTA implementation	• HTA processes and structures in Europe<br>• The role of rapid assessments in the HTA processes<br>• What about effectiveness and cost-effectiveness?<br>• How do countries comparea and are there best practices?<br>• How will JSC and JCA affect national procedures?	• JCAs<br>• PICOs<br>• Modus operandi

***The program of each workshop can be adjusted or changed if needed.**

17:00 - 17:30: Day 1 Wrap-Up and Key Takeaways

Day 2, 12. 2. 2025: Continued Workshops and Networking

8:30 - 9:00: Arrival and registration of participants

9:00 - 15:00: Parallel Workshops (Day 2)

	**Workshop A:** Evidence informed decision making in health care organizations	**Workshop B:** Innovation and Lifecycle Approach (including Early HTA)	**Workshop C:** Rapid assessments	**Workshop D:** HTAR and implementation
**Workshop leader**	**Iga Lipska**	**Inaki Gutierrez-Ibarluzea and Michal Stanak**	**Wim Goetsch and Alric Ruether**	**Marco Marchetti and Aydin Burçak**
9:00 – 10:30 Continued Workshops	Deep dive into specific case studies and outcomes	Exploring challenges and solutions in HTA processes	Practical applications and tools for effective scanning	Medical devices
**10:30 – 11:00 Networking Break**				
11:00 – 12:30 Continued Workshops				
**12:30 – 13:30 Lunch Break**				
13:30 – 15:00 Final Workshops Wrap-UP				

***The program of each workshop can be adjusted or changed if needed.**

15:00 – 15:30: Afternoon Break

15:30 – 16:30: Closing Panel

The Future of HTA and Key Opportunities Ahead

- Panelists:
  - **Andrej Janžič**, MoH Slovenia
  - **Alric Ruether**, IQWIG, Germany
  - **Marko Korenjak**, European Liver Patients' Association, Belgium
  - **Michal Stanak**, NIHO, Slovakia
  - **Anita Milas-Anzić**, MoH Croatia

16:30 – 17:00: Closing Remarks
